# Silent Suffering: Congenital Insensitivity to Pain With Anhidrosis in a Single Family

**DOI:** 10.7759/cureus.115289

**Published:** 2026-08-27

**Authors:** Abdullah Alshehri, Mohaned Mohammed, Khalid Alshehri, Samah E Mohammed, Mohammed Tayalseed, Shady Wafa

**Affiliations:** 1 Pediatric Critical Care Unit, Armed Forces Hospital Southern Region, Khamis Mushait, SAU; 2 Pediatric Medicine, Armed Forces Hospital Southern Region, Khamis Mushait, SAU; 3 Pediatric Intensive Care Unit, Armed Forces Hospital Southern Region, Khamis Mushait, SAU; 4 Pediatrics, Armed Forces Hospital Southern Region, Khamis Mushait, SAU

**Keywords:** autonomic nervous systems, congenital insensitivity to pain with anhidrosis (cipa), hereditary sensory and autonomic neuropathy type iv (hsan iv), joint deformities, recurrent fractures

## Abstract

Hereditary sensory and autonomic neuropathy type IV, commonly known as congenital insensitivity to pain with anhidrosis (CIPA), is a rare autosomal recessive disorder. It is characterized by a profound dysfunction of the sensory and autonomic nervous systems, leading to a global inability to perceive pain or temperature alongside a complete absence of sweating. Clinically, affected individuals typically present with a classic triad of symptoms: severe analgesia, a total lack of pain perception that frequently results in self-mutilation (such as biting the tongue, lips, and fingertips), recurrent fractures, and joint deformities; anhidrosis, which impairs body temperature regulation and often triggers life-threatening hyperpyrexia; and varying degrees of neurological involvement, including intellectual disability and behavioral challenges. This report details a case series within a single extended family involving two sisters and two of their paternal cousins (a brother and sister). All four patients presented with classic clinical hallmarks of CIPA, which were subsequently confirmed through genetic analysis. The patients exhibited multiple complications, including chronic infections and orthopedic injuries resulting from their lack of protective pain sensation. Managing CIPA requires a highly specialized, multidisciplinary approach to mitigate self-injury and manage febrile episodes.

## Introduction

Congenital insensitivity to pain with anhidrosis (CIPA), also classified as hereditary sensory and autonomic neuropathy type IV (HSAN IV), is an exceedingly rare autosomal recessive disorder, with an estimated prevalence of approximately 1 in 125 million live births [1]. The condition is caused by loss-of-function mutations in the *NTRK1* gene, which encodes the receptor tyrosine kinase for nerve growth factor (NGF). Under physiological conditions, the development and survival of nociceptive sensory neurons and sympathetic autonomic neurons are strictly dependent on NGF signaling [2]. Consequently, mutations in *NTRK1* lead to the failed development of dorsal root ganglion neurons, resulting in a total absence of pain and temperature perception. Furthermore, the lack of sympathetic innervation to the sweat glands results in widespread anhidrosis, a hallmark clinical feature of the disorder [3].

The clinical triad of CIPA, including analgesia, anhidrosis, and intellectual disability, presents significant morbidity. The inability to regulate body temperature frequently leads to recurrent hyperpyrexia and febrile seizures [4]. Beyond neurological deficits, the absence of nociceptive fibers in the skeletal system disrupts normal bone metabolism and the differentiation of osteoprogenitor cells. This loss of trophic support often manifests as recurrent fractures, impaired bone consolidation, and joint deformities [5]. While the global literature on CIPA remains limited, case studies are vital for understanding the phenotypic variability of the disease. In this report, we describe a case series of four patients from a single extended family in the Tihama region of southern Saudi Arabia. Diagnosis was established through characteristic clinical presentations and confirmed via genetic analysis.

The patients in this series were from one family and exhibited the classic manifestations of HSAN IV; however, their clinical courses varied significantly, ranging from severe disability to a fatal outcome due to heat stroke.

## Case presentation

**Figure 1:**
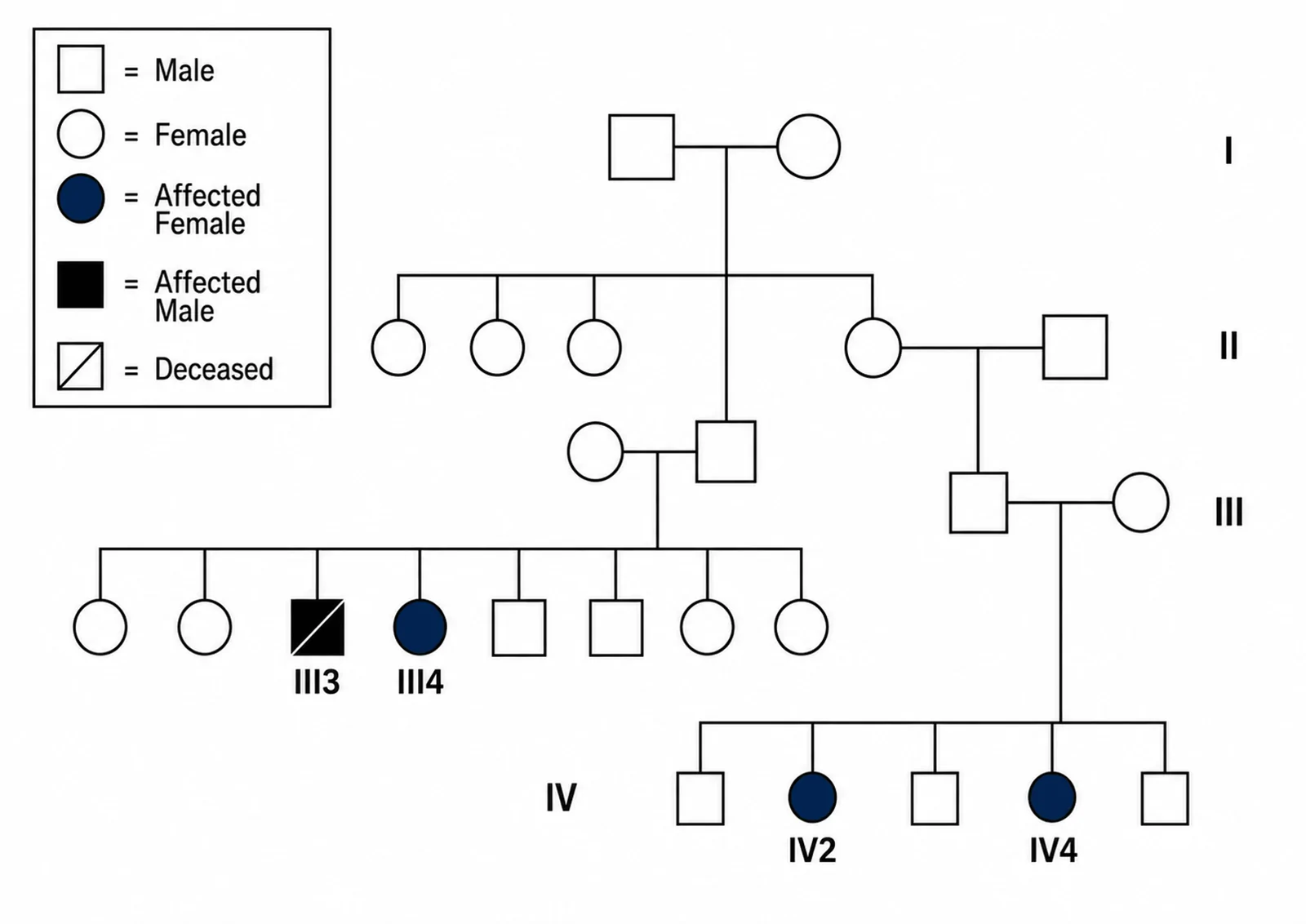
Family pedigree analysis illustrating the familial inheritance pattern. Pedigree analysis demonstrating an autosomal recessive mode of inheritance. The disease skips generations and affects siblings born to clinically unaffected parents, consistent with carrier parents transmitting the mutant allele to affected offspring.

In this case series, we describe the presentation of HSAN IV in four individuals of one family sharing a clinical phenotype, highlighting their presentation, diagnostic tools, and outcomes. The cases were retrospectively reviewed at Armed Forces Hospital South Region (AFHSR), Kingdom of Saudi Arabia, from 2024 to 2026. All eligible cases identified during the study period were included consecutively. A pedigree analysis was performed to illustrate the familial inheritance pattern (Figure 1).

Case 1

**Figure 2:**
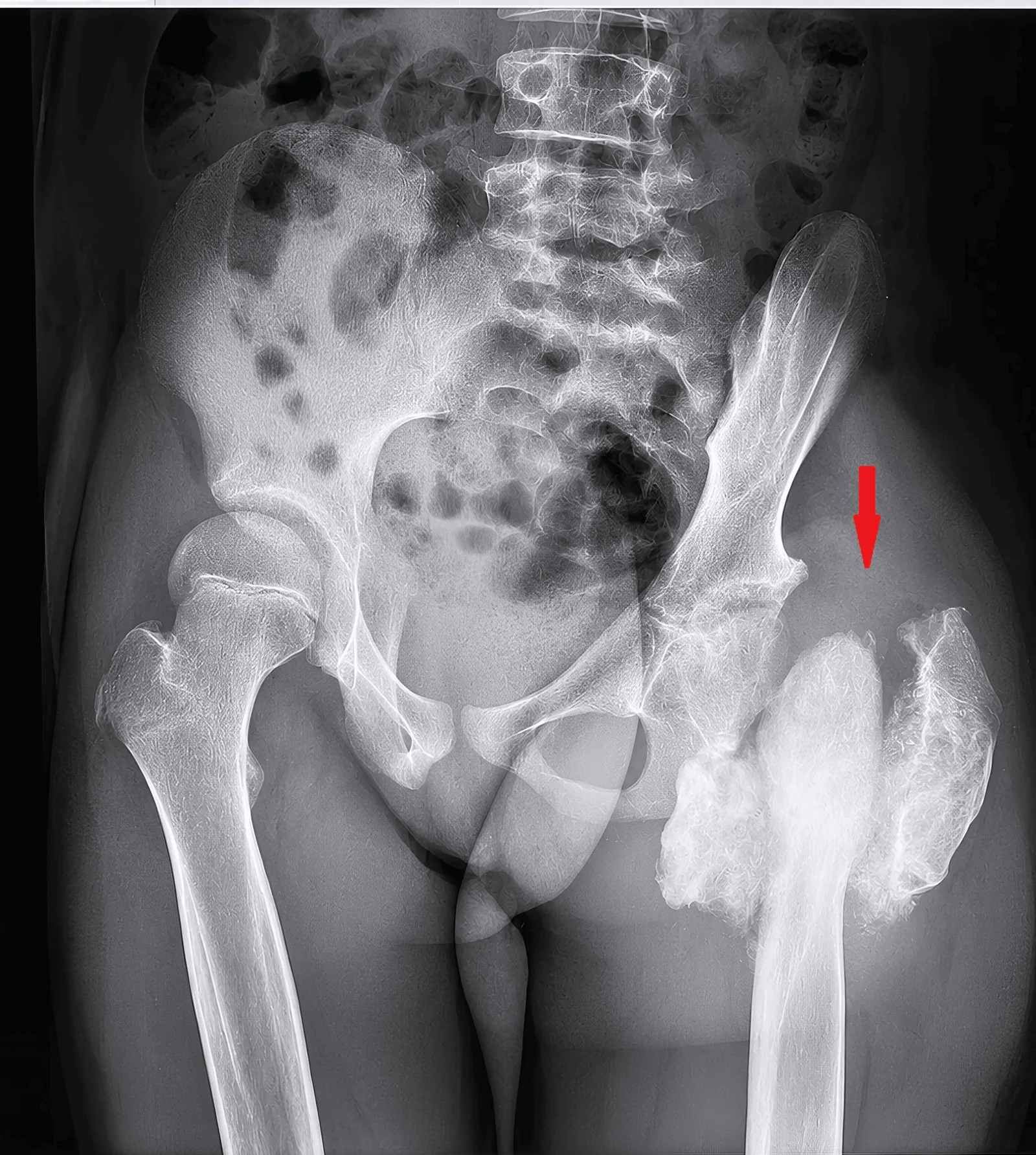
The left hip region showing a very large, dense, irregular calcified mass around the upper femur and hip.

**Figure 3:**
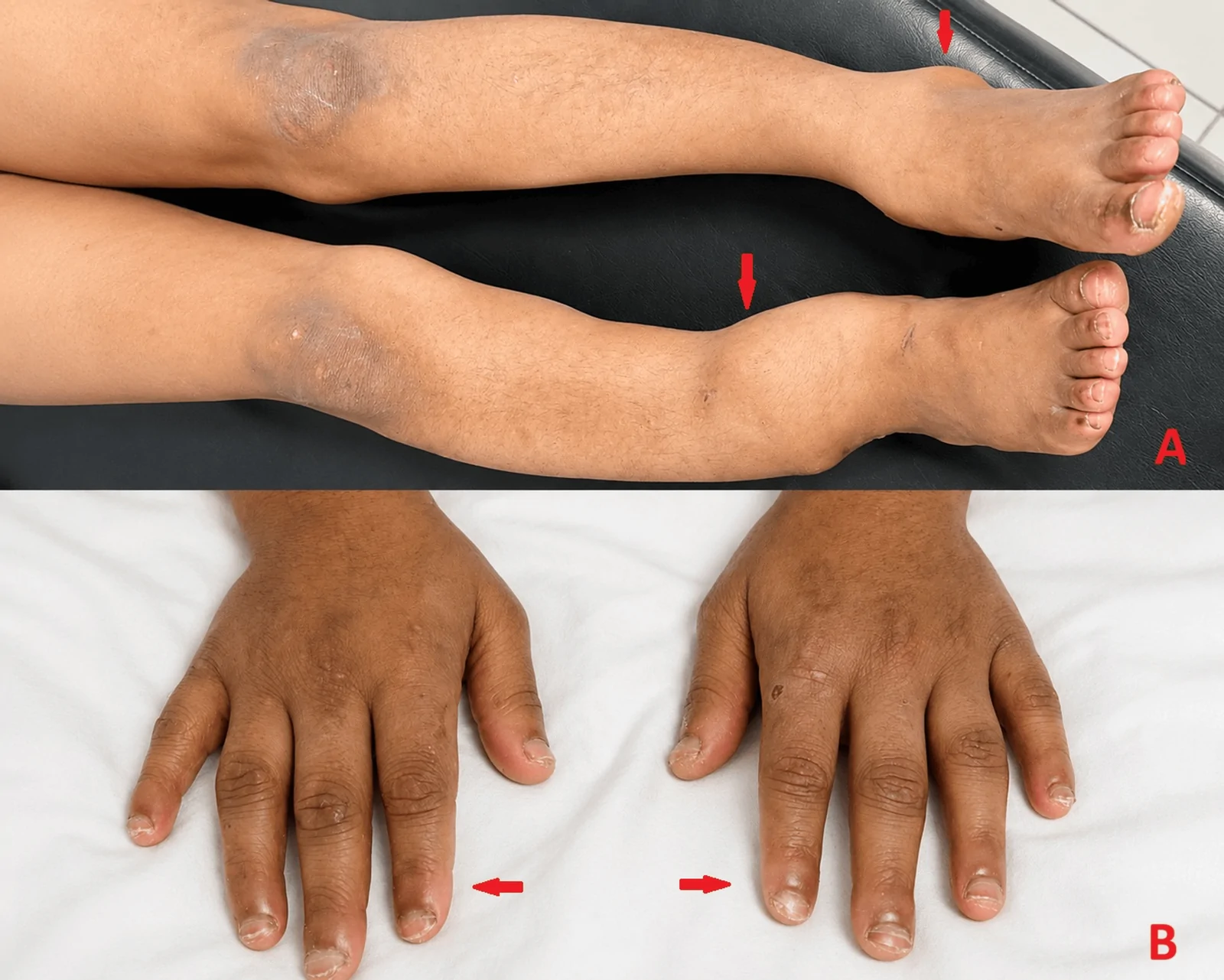
Clinical manifestations of chronic painless skeletal trauma. (A) Lower extremities demonstrating severe, bilateral tibial deformities, exuberant bony remodeling, and localized swelling (red arrows) indicative of neuroarthropathy (Charcot joints) and malunited fractures. (B) Dorsal view of the hands showing shortened, thickened digits with distal phalangeal remodeling (red arrows) and diffuse cutaneous scarring from recurrent, painless microtrauma and self-injurious behavior.

An 11-year-old (IV4) female born to a consanguineous marriage was diagnosed at the age of five years with CIPA via genetic testing, which was further supported by two paternal cousins with the same genetically confirmed condition (Figure 1). Her clinical journey began in early infancy when her parents observed a complete lack of pain response during routine vaccinations, which was soon followed by recurrent, unexplained febrile seizures by one year of age. Over subsequent years, she suffered multiple painless fractures, most notably involving the left ankle. At five years of age, she required surgical fixation for a right tibial fracture, which was later complicated by delayed union as well as osteomyelitis of the left leg and hip joint (Figure 2) that necessitated intensive antibiotic therapy. She also presented with a significant limb length discrepancy alongside residual deformities of the right leg, left thigh, and left ankle (Charcot joint) (Figure 3A). Despite these severe orthopedic and infectious setbacks, she achieved all neurodevelopmental milestones on schedule and is currently thriving academically in the fourth grade at a regular school. On physical examination, she exhibited failure to thrive with both weight (22 kg) and height (121 cm) falling below the third percentile; generalized xeroderma over her hands, knees, and feet; and evident finger-biting marks from self-mutilation (Figure 3B). Neurologically, her motor examination was unrevealing with preserved muscle bulk, tone, power, and deep tendon reflexes. Sensory evaluation demonstrated a complete absence of withdrawal responses to painful or thermal stimuli across her face, trunk, and extremities alongside absent corneal reflexes. Proprioception, position sense, and tear production remained fully intact. Nerve conduction studies demonstrated normal motor and sensory nerve conduction velocities and amplitudes, with no electrophysiological evidence of peripheral neuropathy. Finally, her dysautonomia manifested as prominent generalized anhidrosis, clinically confirmed by an inability to perspire even during high-grade fevers. Whole-exome sequencing (WES) confirmed the genetic defect in *NTRK1* (variant *c.1426A>T*). Despite the thermoregulatory instability, she maintains cardiovascular stability with normal blood pressure and heart rate profiles, with no signs of acute autonomic crisis. Her laboratory investigations were within normal ranges (Table 1).

**Table 1:** Laboratory findings. WBC = white blood cells; ALT = alanine aminotransferase; AST = aspartate aminotransferase; ALP = alkaline phosphatase

Laboratory test	Result	Units	Reference range
WBC	6.9	×10^9^/L	4.5–13.5
Hemoglobin	12.8	g/dL	10.9–15
Platelet	298	×10^9^/L	150–450
Renal function test			
Urea	4.3	mmol/L	2.9–7.1
Creatinine	42	μmol/L	57–113
Serum sodium	139	mmol/L	136–144
Serum potassium	4.2	mmol/L	3.6–5.1
Liver function test			
ALT	21	U/L	11–39
AST	24	U/L	22–58
ALP	245	U/L	110–341
Total bilirubin	8	μmol/L	<34
Albumin	43	g/L	35–50
Total protein	72	g/L	56–77
Bone profile			
Calcium	2.35	mmol/L	2.23–2.58
Phosphate	1.58	mmol/L	0.78–1.53
Magnesium	0.86	mmol/L	0.66–1.07

Case 2

**Figure 4:**
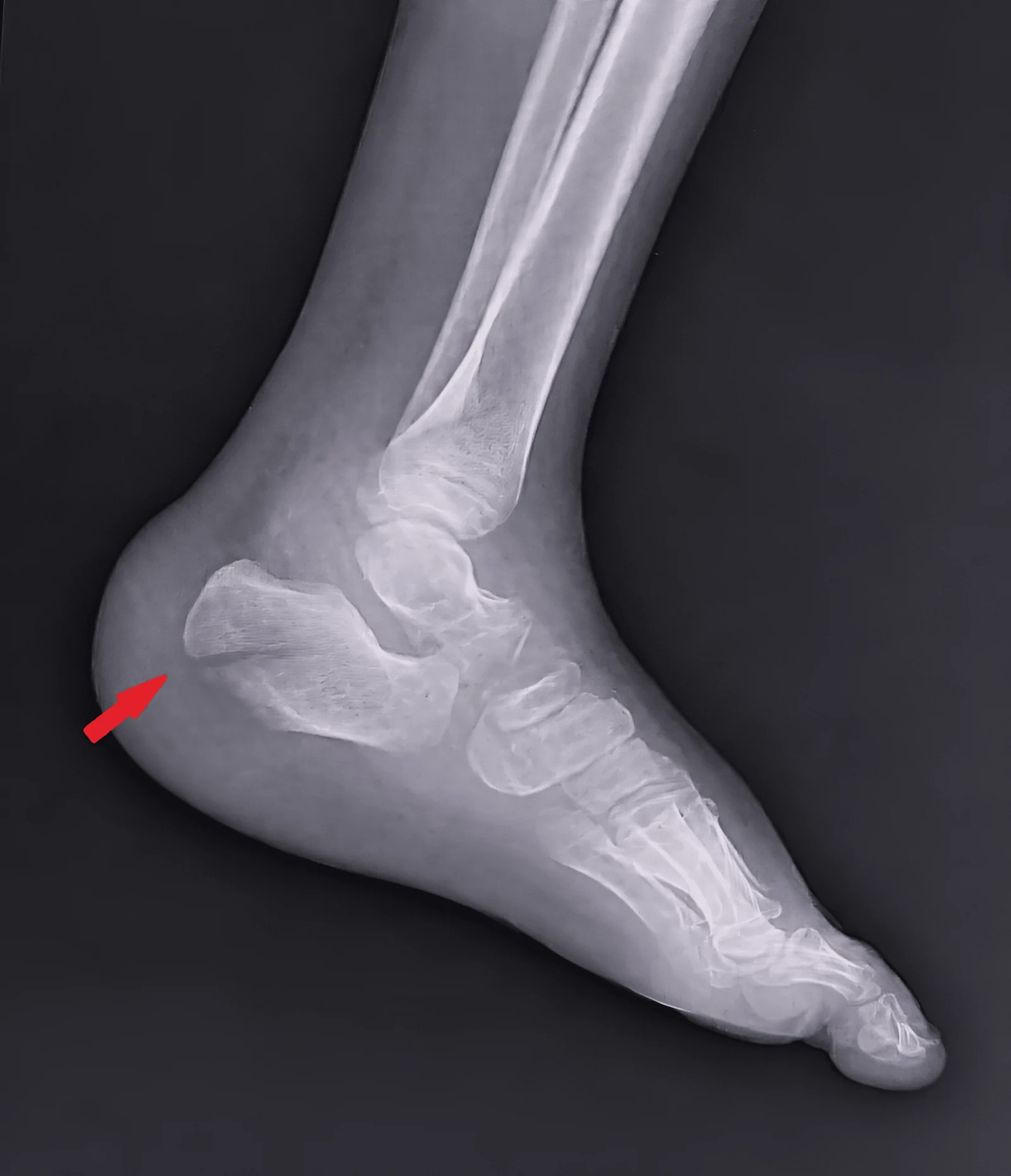
Lateral view of the foot and ankle. The ankle, hindfoot, and midfoot showing a very high arch and plantar-flexed forefoot pictures of chronic contracture and deformity of the hindfoot/midfoot with fructure calcaneus.

A five-year-old (IV4) female, the youngest sister of the previously described affected female, was born at term via cesarean section. She was born into a family with a known history of CIPA. While her three brothers were clinically asymptomatic, she shared this condition with an older sister. Genetic testing at two years of age formally confirmed her diagnosis. Her clinical journey began in early infancy. Her parents first noticed a profound lack of response to painful stimuli during routine vaccinations. This was accompanied by recurrent episodes of unexplained high-grade fevers and a marked inability to sweat. By the age of one year, these severe thermoregulatory challenges manifested as recurrent febrile seizures, requiring medical intervention. Developmentally, the patient exhibited global developmental delay, characterized by intellectual disability and significant speech delay. Her condition was further complicated by self-injurious behavior, specifically repetitive nail-biting that left visible marks on her fingers. Her medical history was also notable for a serious left foot injury. What began as a localized calcaneus (heel bone) injury eventually progressed to a calcaneal fracture (Figure 4) complicated by osteomyelitis. This was successfully managed with intravenous antibiotic therapy, leading to a complete clinical recovery. During her physical examination, the patient displayed characteristic dermatological findings of CIPA, including thickened, dry skin (xeroderma) over her hands, knees, and feet. A neurological examination revealed normal muscle bulk, tone, and power, with preserved deep tendon reflexes. While her large-fiber sensory modalities, including vibration and joint position sense, were fully preserved, a targeted sensory examination confirmed a complete loss of pain and temperature sensation over her face, trunk, and extremities, with zero response to pinprick or thermal stimuli. Finally, a formal cognitive assessment revealed an intelligence quotient (IQ) of 61, confirming a mild intellectual disability. WES confirmed the genetic defect in *NTRK1* (variant *c.1426A>T*). Her laboratory investigations were within normal ranges (Table 2).

**Table 2:** Laboratory findings. WBC = white blood cells; ALT = alanine aminotransferase; AST = aspartate aminotransferase; ALP = alkaline phosphatase

Laboratory test	Result	Units	Reference range
WBC	7.8	×10^9^/L	4.5–13.5
Hemoglobin	12.4	g/dL	10.9–15
Platelet	315	×10^9^/L	150–450
Renal function test			
Urea	10	mmol/L	2.9–7.1
Creatinine	50	μmol/L	57–113
Serum sodium	137	mmol/L	136–144
Serum potassium	4.9	mmol/L	3.6–5.1
Liver function test			
ALT	21	U/L	11–39
AST	43	U/L	22–58
ALP	321	U/L	110–341
Total bilirubin	5	μmol/L	<34
Albumin	39	g/L	35–50
Total protein	70	g/L	56–77
Bone profile			
Calcium	2.00	mmol/L	2.23–2.58
Phosphate	1.6	mmol/L	0.78–1.53
Magnesium	0.84	mmol/L	0.66–1.07

Currently, both sisters remain in stable condition under the care of a comprehensive multidisciplinary team, including specialists in orthopedics, dermatology, neurology, and general pediatrics. Management is primarily supportive and preventive, focusing on rigorous injury prevention, strict environmental temperature control to minimize the risk of hyperthermia, and prompt treatment of wounds to prevent deep-seated infections. She continues to receive regular physiotherapy and developmental assessments to optimize her functional outcomes

Case 3

A 37-year-old male (III3) and the first cousin of the previously described patients was born at term via spontaneous vaginal delivery following an uneventful pregnancy. He held a significant place in his family’s medical history as the first individual to receive a genetic diagnosis of CIPA. His clinical journey began in early infancy with persistently elevated body temperatures and a total absence of sweating. These severe thermoregulatory failures led to recurrent febrile seizures of initially unknown origin, resulting in frequent hospital admissions throughout his early years. As he transitioned into early childhood, classic behavioral markers of CIPA emerged, most notably self-injurious habits such as repetitive lip-biting. Based on these early manifestations, CIPA was strongly suspected, and the diagnosis was subsequently confirmed through genetic testing. Unlike his younger cousins, this patient’s clinical course was predominantly defined by severe and progressive orthopedic complications. Lacking protective pain sensation, he sustained multiple recurrent, painless fractures across several major bones, including his humerus, femur, and both tibiae. Surgical interventions were needed due to the inherent instability of the fractures and a persistent tendency toward delayed bone healing, with several injuries requiring surgical fixation. His mobility and recovery were further complicated by recurrent episodes of osteomyelitis, which necessitated prolonged and intensive courses of intravenous antibiotic therapy. Remarkably, despite the severe physical trauma and the immense physiological toll of his condition, his cognitive development and developmental milestones remained entirely within normal limits. This preserved intellect distinctly set his clinical presentation apart from the global developmental delays observed in other family members.

Tragically, at the age of 37, the patient passed away due to severe heat stroke. His outcome serves as a sobering clinical reminder of the lethal, life-threatening risks posed by the profound thermoregulatory impairment that defines this rare genetic disorder.

Case 4

A 39-year-old female (III4) was the youngest sister of the previously described affected male. She was one of eight siblings, four of whom, along with three brothers, remained clinically unaffected by the condition. Her medical journey began at one year of age when she presented with recurrent febrile seizures and episodes of unexplained hyperthermia, which were later linked to a marked inability to sweat. By the age of four years, the characteristic absence of protective pain sensation became starkly evident after she began sustaining painless fractures in both tibiae. Given her older brother’s known health history, targeted genetic analysis was performed. Testing revealed mutations in the *NTRK1* gene, confirming a diagnosis of CIPA. Interestingly, while her genetic profile was definitive, concurrent nerve and muscle biopsies yielded entirely unremarkable results. Over the following decades, the patient’s clinical course was heavily dominated by significant orthopedic morbidity. Deprived of normal pain cues, she sustained numerous recurrent fractures across her hand, femur, and both lower limbs. These repeated skeletal traumas combined with a complete lack of sensory feedback eventually triggered the development of Charcot arthropathy, a progressive, debilitating degeneration of her weight-bearing joints. On physical examination, her chronic skeletal complications were readily visible as notable deformities across both knees and lower limbs. Consistent with the anhidrotic nature of CIPA, her skin remained dry and thickened (xeroderma) over her fingers, knees, and feet. Neurological examination showed almost the same picture as previous patients, as she maintained normal muscle tone, power, and preserved deep tendon reflexes. However, targeted sensory testing revealed a profound, complete loss of pain and temperature perception spanning her face, trunk, and all four extremities. WES confirmed the genetic defect in *NTRK1* (Variant *c.1426A>T*). Due to the severe, cumulative destruction of her joints, the patient now requires physical assistance with ambulation. She remains under the intensive care of a broad multidisciplinary team, including specialists in rheumatology, neurology, dermatology, and physiotherapy. Her regular laboratory follow-up was within the normal range (Table 3). Her current plan of care centers on strict preventive strategies, most notably maintaining precise environmental temperature control to avoid dangerous hyperthermia, along with tailored lifestyle modifications to prevent further fractures and skin breakdown.

**Table 3:** Laboratory findings. WBC = white blood cells; ALT = alanine aminotransferase; AST = aspartate aminotransferase; ALP = alkaline phosphatase

Laboratory test	Result	Units	Reference range
WBC	9.3	×10^9^/L	4.5–13.5
Hemoglobin	14.2	g/dL	10.9–15
Platelet	462	×10^9^/L	150–450
Renal function test			
Urea	2.2	mmol/L	2.9–7.1
Creatinine	23	μmol/L	57–113
Serum sodium	141	mmol/L	136–144
Serum potassium	4.2	mmol/L	3.6–5.1
Liver function test			
ALT	26	U/L	11–39
AST	31	U/L	22–58
ALP	245	U/L	110–341
Total bilirubin	59	μmol/L	<34
Albumin	37	g/L	35–50
Total protein	72	g/L	56–77
Bone profile			
Calcium	2.4	mmol/L	2.23–2.58
Phosphate	1.31	mmol/L	0.78–1.53
Magnesium	0.9	mmol/L	0.66–1.07
Vitamin D	36	ng/mL	20–70

A comparative summary of the four cases is presented in Table 4, highlighting the key clinical features and the most striking differences in disease presentation, complications, and outcomes.

**Table 4:** Comparative clinical, neurological, orthopedic, genetic, and outcome characteristics of the four affected family members.

Characteristic	Case 1	Case 2	Case 3	Case 4
Age/Sex	11-year-old female	5-year-old female	37-year-old male	39-year-old female
Family relationship	Affected younger-generation female; paternal cousins also affected	Younger sister of Case 1	First cousin; first affected family member to receive genetic diagnosis	Younger sister of Case 3
Age at diagnosis	5 years	2 years	Childhood	Childhood
Consanguinity	Born to consanguineous marriage	Same family	Same family	Same family
Loss of pain sensation	Complete	Complete	Profound	Complete
Loss of temperature sensation	Complete	Complete	Present	Complete
Anhidrosis/thermoregulatory dysfunction	Generalized anhidrosis with recurrent hyperthermia	Marked anhidrosis with recurrent high-grade fever	Severe thermoregulatory dysfunction with recurrent febrile episodes	Anhidrosis with recurrent hyperthermia
Febrile seizures	Present since infancy	Present since 1 year	Recurrent during childhood	Present since 1 year
Cognitive/Developmental status	Normal development; academically thriving	Global developmental delay; IQ 61	Normal cognitive development	No developmental impairment reported
Self-injurious behavior	Finger biting	Nail biting	Lip biting	Not specifically reported
Major orthopedic manifestations	Multiple painless fractures, delayed union, osteomyelitis, limb-length discrepancy, Charcot joint and limb deformities	Calcaneal fracture complicated by osteomyelitis	Multiple recurrent fractures involving humerus, femur and both tibiae; delayed healing	Multiple recurrent fractures involving hand, femur and lower limbs; severe Charcot arthropathy
Osteomyelitis	Present; multiple sites	Present; left foot	Recurrent	Not specifically reported
Charcot arthropathy	Present	Not reported	Not reported	Severe and progressive
Skin manifestations	Xeroderma and scars from recurrent trauma	Xeroderma and self-trauma marks	Not specifically reported	Xeroderma
Nerve conduction studies	Normal motor and sensory studies	Not reported	Not reported	Not reported
Nerve/Muscle biopsy	Not reported	Not reported	Not reported	Unremarkable
Genetic finding	*NTRK1* c.1426A>T	*NTRK1* c.1426A>T	CIPA confirmed genetically; specific variant not stated	*NTRK1* c.1426A>T
Cardiovascular/Autonomic status	Cardiovascular stability despite dysautonomia	Not specifically reported	Severe thermoregulatory dysfunction; fatal heat stroke	Not specifically reported
Functional status/Outcome	Attending regular school and thriving academically	Stable with multidisciplinary care	Died at age 37 from severe heat stroke	Requires physical assistance with ambulation

## Discussion

CIPA is a rare autosomal recessive disorder primarily caused by loss-of-function mutations in the *NTRK1* gene. Located on chromosome 1q23, this gene comprises 17 exons and encodes the high-affinity receptor for NGF [6]. To date, over 100 distinct mutations, predominantly missense or nonsense variants, have been identified. These mutations typically impair the protein’s intracellular tyrosine kinase domain, which effectively halts critical NGF signaling pathways [7]. The hallmark loss of pain and temperature sensation in CIPA patients stems from the developmental failure of small-diameter sensory neurons. Under normal physiological conditions, these neurons express the *NTRK1* protein to receive survival signals from NGF [8]. In the absence of this signaling, these cells undergo apoptosis during embryonic development or early infancy. This cellular loss results in a severely reduced density of the sensory nerve fibers responsible for transmitting nociceptive and thermosensitive stimuli from the periphery to the central nervous system [9].

The clinical presentation of these four cases aligns closely with the established global literature, yet provides critical insights into the long-term natural history of the disorder. Compared to the Japanese series reported by Indo et al., which characterized over 100 distinct *NTRK1* mutations, the patients in this series demonstrate the classic “Tihama phenotype” where environmental factors, specifically the extreme heat of southern Saudi Arabia, exacerbate the inherent thermoregulatory failure of the disease [2]. While global literature often focuses on the early childhood presentation of self-mutilation, these cases highlight the secondary orthopedic complications that dominate the clinical course of adults. The development of Charcot arthropathy and chronic osteomyelitis seen in Case 4 is consistent with the findings of Bar-On et al. [10], who noted that CIPA patients develop significant joint deformities by the second decade of life due to the lack of “trophic” neuroskeletal signaling. Interestingly, while literature by Shatzky et al. [2] suggests that a majority of CIPA patients exhibit some degree of intellectual disability, Cases 1 and 3 in this series achieved normal cognitive milestones. This supports the emerging hypothesis that phenotypic expression of *NTRK1* mutations may be more heterogeneous than previously thought, perhaps influenced by the specific location of the mutation within the tyrosine kinase domain.

Furthermore, the fatal outcome in Case 3 at age 37 provides a rare but critical data point on adult mortality. Literature on CIPA mortality is heavily skewed toward early childhood, with many sources citing a 20% mortality rate before age three due to hyperpyrexia. However, this case series mirrors the findings of Yagev et al. [11], who demonstrated that the risk of fatal hyperthermia remains a lifelong threat. By situating these cases within the broader literature, it becomes clear that while the genetic etiology is singular, the clinical trajectory is heavily dictated by a combination of specific mutational severity and the rigor of the surrounding multidisciplinary care. Clinically, CIPA must be distinguished from several overlapping conditions. It belongs to the broader category of HSAN, which includes eight distinct types differentiated by their specific genetic mutations. It is notably different from congenital indifference to pain (CIP); while those with CIP can physically perceive pain and temperature, they lack the typical emotional or behavioral distress associated with it and do not suffer from anhidrosis [12].

Because there is currently no cure, management is strictly supportive and multidisciplinary. Primary care focuses on rigorous thermoregulation to prevent life-threatening hyperpyrexia and febrile seizures, utilizing antipyretics and active cooling measures. Protective strategies are essential to minimize self-mutilating behaviors and unnoticed trauma, which can lead to sepsis or chronic orthopedic issues. Regular dental and ophthalmological evaluations are also vital to prevent oral trauma and corneal ulcerations [13].

## Conclusions

CIPA is a rare genetic disorder that necessitates a vigilant, multidisciplinary approach to prevent life-threatening complications. While current management focuses on supportive care and injury prevention, the profound burden on patients highlights a critical need for early screening and the development of advanced genetic and stem cell therapies. Ultimately, timely intervention and scientific innovation are essential to improving the long-term clinical outcomes and quality of life for those living with this complex condition.
